# Overcoming prevalence bias in colorectal cancer screening studies by sensible analyses rather than ignoring it or giving up relevant effect measures

**DOI:** 10.1007/s10654-025-01319-5

**Published:** 2025-11-08

**Authors:** Hermann Brenner, Thomas Heisser, Michael Hoffmeister

**Affiliations:** 1 Cancer Prevention Graduate School, German Cancer Research Center (DKFZ), INF 280, 69120 Heidelberg, Germany; 2 Division of Clinical Epidemiology of Early Cancer Detection, German Cancer Research Center (DKFZ), Heidelberg, Germany

**Keywords:** Bias, Colorectal cancer, Incidence, Prevalence, Randomized trial, Screening

We thank Drs. Haug et al. [1] and Piccininni et al. [2] for their interest in our critical view [3–6] on the published analysis of the preliminary NordICC trial results [7].

The comments by Haug et al. help to make one of the major limitations of the design, analysis and interpretation of that trial even clearer: Screening colonoscopy exerts beneficial effects not only by preventing incident colorectal cancer (CRC), but also by early-detecting prevalent CRC. Both components make major contributions to lowering the burden of CRC, such as type and costs of CRC treatment, disease related and treatment related restrictions in quality of life and CRC mortality [8–11]. The „incidence“ metrics aimed for in the design and derived in the analysis of the NordICC trial count early-detected prevalent cases on the „negative side“ of outcomes, by lumping them together with the non-prevented incident cases. We coined the term „prevalence bias“ to reflect this major limitation, because early detection is one aim of screening and should not be counted on the negative side. However, beyond the concern on semantic imprecision of lumping prevalence and incidence in the NordICC trial report, the much more important concern is that failure to distinguish prevalent and incident cases has serious clinical and practical implications. Ignoring the beneficial effects of early detection of prevalent CRC should not be the basis for decision making, as claimed by Haug et al., neither for health-policy makers nor for individual persons. Such decision making should not be based on misleading calculations of the proportions of prevented „incident“ cases, where “incident cases” in reality are a mixture of non-preventable prevalent and incident CRC cases.

Haug et al. argue that the NordICC trial aimed to answer and actually answered „Research question A“: What is the effectiveness of colonoscopy on CRC incidence in persons eligible for screening. Apart from failure to distinguish prevalent and incident cases in the wording of the aim and the measured outcome, just aiming to disclose some proportion of the screening effects is unduly and unnecessarily unambitious. As illustrated in our work [5, 6], disclosure of both, prevention of incident cases and early detection of prevalent cases is possible and should be aimed for and reported. In the NordICC trial, 10-year CRC risk was 0.98% in the invited group and 1.20% in the usual-care group. As these percentages include cases that were already prevalent at baseline and no longer preventable by screening, the reported relative risk of 0.98/1.20 = 0.82, corresponding to a risk reduction by 18%, is misleading and incompletely reflecting the screening effects.

However, under the plausible assumption that (at least the vast majority of) prevalent CRC have become clinically manifest within the 10-year follow-up period, the risk difference, 1.20%−0.98%=0.22% may still represent a valid, albeit incomplete metric of the effects of the screening offer among the 28,220 invited participants. A more comprehensive quantification of the effects should additionally consider earlier detection of prevalent cases. From the NordICC trial report it can be derived that prevalent CRC was detected at screening colonoscopy (and thereby earlier detected than it would have been detected otherwise) among 62 out of 28,220 invited participants, which corresponds to an early detection proportion of 62/28,220 = 0.22%. As illustrated by our work [5, 6], just restricting the effect measure to the 0.22% cases of prevented incident CRC and ignoring the 0.22% early-detected prevalent CRC provides a very incomplete representation of the screening effects on the population level, given that earlier detection of CRC goes along with less invasive and less costly treatment, better quality of life and better prognosis.

Haug et al. claim that that their stated Research question A would also be the most relevant to support decision making of individuals. However, from the individual perspective, like from the public health perspective, it is inappropriate to ignore early detection effects of screening in decision making. More relevant research questions to support decision making of individuals than those stated by Haug et al. would be, for example,

(i) How likely will screening colonoscopy early-detect a possible prevalent but yet undetected CRC?
(ii) How likely will screening colonoscopy prevent an incident CRC by early-detection and removal of precancerous lesions?

Answers to these questions can and should likewise be more validly be obtained by alternative metrics, as illustrated in our work [5, 6]. For example, while it is safe to assume that screening colonoscopy early-detects prevalent CRC with a sensitivity of close to 100%, our re-analysis of the reported NordICC data suggest that it additionally prevents approximately 60% of the incident CRC. As explained in our work [6], this proportion is still underestimated due to other limitations of the NordICC trial methodology.

Regarding the hypothetical example presented by Haug et al., the relevant information, disclosed by the type of analyses we propose, would be that screening would early-detect all prevalent cases and prevent all incident cases, a more comprehensive and relevant message than the shortcut and misleading message stated by Haug et al. that screening would reduce „incidence“ by just 20%. As demonstrated by our work, such relevant information could be derived by straightforward calculations, without any need of the unethical study design outlined in Fig. 1 by Haug et al.

Piccininni et al. [2] call for making prevalence bias irrelevant by restricting reported incidence-related effect metrics to incidence differences and abstaining from reporting reduction of incidence in relative terms. This would be a paradigm shift of reporting indeed, given that only relative effect measures, but no absolute effect measures have been reported as main results in the abstracts of the NordICC trial report [7] and the reports on most of the landmark trials on the effects of screening sigmoidoscopy [12–18]. However, there have been good reasons for the priority given to relative over absolute effect measures: They are much easier to communicate to and understand by a broad audience including the target population of CRC screening, and they are much more stable across a wide range of key determinants, such as length of follow-up or age. As demonstrated in our work [10, 11], there is no need to give up relevant and well-communicable relative effect metrics in order to minimize prevalence bias (which is smaller, but still present in the absolute effect metrics favoured by Piccininni et al. [2]). The easy-to-derive metrics proposed in our work enable overcoming prevalence bias, disentangling early-detection and prevention effects and reporting relevant effect measures in a semantically and content-wise valid, easy-to-communicate manner.

In summary, rather than ignoring the presence of prevalence bias [1] or giving up relevant and well-communicable effect measures [2], sticking to misleading analyses and interpretations of the NordICC trial and preserving prevalence bias in emulated target trial approaches [19], we strongly encourage application of analyses that provide more valid, more precise and more relevant information for both public health and individual persons‘ decision making. Misinterpretations of the NordICC trial results have already done tremendous harm by casting reluctance in engagement for CRC screening. Well-organized CRC screening, for which screening colonoscopy is just one of several screening exam options, is one of the most powerful approaches to CRC control, as demonstrated since decades by compelling evidence from observational studies [20, 21], modelling studies [22] and real-world evidence of trends in CRC incidence and mortality [23, 24]. Rectifying misinterpretation of the NordICC trial results is not just an academic exercise but should be a public health priority.
